# An Efficient and Low-Complexity Transformer-Based Deep Learning Framework for High-Dynamic-Range Image Reconstruction

**DOI:** 10.3390/s25051497

**Published:** 2025-02-28

**Authors:** Josue Lopez-Cabrejos, Thuanne Paixão, Ana Beatriz Alvarez, Diodomiro Baldomero Luque

**Affiliations:** PAVIC Laboratory, University of Acre (UFAC), Rio Branco 69915-900, Brazil; thuannepaixao@gmail.com (T.P.); ana.alvarez@ufac.br (A.B.A.); baldomero.luque@ufac.br (D.B.L.)

**Keywords:** high-dynamic-range imaging, image reconstruction, channel attention, spatial attention, Transformers

## Abstract

High-dynamic-range (HDR) image reconstruction involves creating an HDR image from multiple low-dynamic-range images as input, providing a computational solution to enhance image quality. This task presents several challenges, such as frame misalignment, overexposure, and motion, which are addressed using deep learning algorithms. In this context, various architectures with different approaches exist, such as convolutional neural networks, diffusion networks, generative adversarial networks, and Transformer-based architectures, with the latter offering the best quality but at a high computational cost. This paper proposes an HDR reconstruction architecture using a Transformer-based approach to achieve results competitive with the state of the art while reducing computational cost. The number of self-attention blocks was reduced for feature refinement. To prevent quality degradation, a Convolutional Block Attention Module was added, enhancing image features by using the central frame as a reference. The proposed architecture was evaluated on two datasets, achieving the best results on Tel’s dataset in terms of quality metrics. The computational cost indicated that the architecture was significantly more efficient than other Transformer-based approaches for reconstruction. The results of this research suggest that low-complexity Transformer-based architectures have great potential, with applications extending beyond HDR reconstruction to other domains.

## 1. Introduction

Capturing information faithfully from a scene using conventional cameras is a complex task. Unlike human eyes, a camera compresses the high dynamic range (HDR) of the scene to capture and display it in a low-dynamic-range (LDR) image [1]. This not only limits the luminance range in the captured image but also causes information outside of this range not to be captured in the final image [2]. To address this, specialized hardware has been developed to directly produce an HDR image; however, these devices tend to be very expensive for mass adoption [3,4].

Initially, methodologies based on conventional algorithms were developed to convert LDR images into HDR images, using algorithms to address issues related to object movement in the scene [5,6,7,8]. Other approaches focus on removing defects caused by camera movement [9,10,11,12], and some algorithms employ a patch-based methodology to align the input images [13,14,15]. In recent years, algorithms to convert a single LDR image into an HDR image have been developed. Some methodologies rely on inverse tone mapping [16], while other architectures use deep learning algorithms based on convolutional neural networks (CNNs) [17]. However, results using only a single LDR input image exhibit incomplete information, which can be visually apparent.

With this challenge in mind, a common methodology to maintain the fidelity of the original scene is to capture a series of images at different exposures and subject them to a fusion process, thereby reconstructing the scene with the highest possible fidelity and obtaining an HDR image [13,18]. This approach has particular characteristics. The ideal case is when three images with different exposures are captured at the same moment in time, in which case the only difference between the images is the exposure value. In this scenario, the images would not suffer from misalignment, and consequently, no artifacts would be generated in the HDR reconstruction. In typical scenarios, the images are captured consecutively, making them prone to various types of degradation, such as misalignment, motion, noise, and overexposure, all of which must be corrected during the HDR reconstruction process [19,20]. An approach currently used to perform this reconstruction is deep learning. Some methods employ alignment mechanisms between frames before image fusion [21,22], while others use temporal or spatial attention mechanisms to highlight regions within the frames and then perform the necessary fusion to reconstruct the HDR image [23,24,25]. In both contexts, an image restoration architecture is used to perform the frame fusion and handle the degradation. Typically, conventional methods such as CNN-based architectures are used for this purpose [26,27,28], while more recent approaches employ diffusion-based architectures [29], Generative Adversarial Networks (GANs) [30], and Transformers [31,32].

Regarding Transformers, this architecture was initially used for natural language processing (NLP), being a complete architecture with an encoder–decoder approach, utilizing self-attention to capture long-range dependencies within the processed text [33]. Its domain of use was quickly expanded to include image-related tasks, where only the encoder portion of NLP Transformers was retained. The functioning of this encoder component begins by dividing the image into small patches, which are treated as linear vectors and encoded using position embeddings to retain information about the positions of these patches relative to the original image. These patches are then fed into a series of encoder blocks composed of layer normalization (LN), self-attention, LN, and a multilayer perceptron (MLP). After this process, the patches are reassembled according to their original positions to maintain the original resolution [34].

Transformer-based architectures incur a high computational cost [35] due to the recursive application of self-attention across the entire image [34], resulting in quadratic complexity. This complexity is offset by their powerful encoding capability, which improves state-of-the-art performance in HDR reconstruction tasks. However, as the architecture becomes more complex, the computational cost increases significantly. Consequently, current works in HDR reconstruction focus on achieving the best results in terms of quality metrics of the reconstructed image compared to the original, such as the Peak Signal-to-Noise Ratio (PSNR) or the Structural Similarity Index Measure (SSIM) [36], often overlooking improvements in memory consumption, decreases in processing time, or reductions in the number of parameters [37]. Among Transformer-based HDR reconstruction approaches, SCTNet, proposed by Tel. et al. [32], stands out, as it employs self-attention and cross-attention mechanisms to enhance the features extracted from the input image, achieving the best quality results in the state of the art. When comparing the computational cost of SCTNet with those of previous Transformer-based proposals, a slight improvement is observed because other architectures continue to present high inference times. However, when comparing inference time between SCTNet and other approaches, such as GANs, the SCTNet approach demonstrates inferior results, highlighting the fact that this architecture was not developed with the goal of reducing computational cost.

To maintain good results in terms of quality metrics and improve computational cost, a new HDR reconstruction architecture based on Transformers is proposed. This architecture is based on the SCTNet methodology, where the typical number of self-attention blocks in Transformers is reduced from twenty-four to six self-attention blocks, while two refinement blocks called Convolutional Block Attention Modules (CBAM) blocks [38] are added. The CBAM blocks consist of channel-level attention followed by spatial-level attention, referencing the central exposure image and performing cross-attention with an image of different exposure. The efficiency of this architecture in terms of reconstruction quality is demonstrated by the good quantitative results obtained with the PSNR, SSIM, and HDR-VDP-2 metrics, as well as the achieved qualitative results. The experiments were conducted using the datasets from Kalantari et al. [39] and Tel et al. [32]. Additionally, the results regarding computational cost, calculated from the numbers of parameters and multiply–accumulate units; the model size; and the inference time, are important to highlight, as they demonstrate that the proposed architecture is faster than other Transformer-based models. Thus, the main contributions of this paper can be summarized as follows:

- We propose a new high-quality HDR reconstruction architecture based on Transformers that has a lower computational cost than current state-of-the-art methodologies.
- We introduce the use of CBAM blocks to enhance input features in the self-attention mechanisms of the Transformer architecture for HDR reconstruction.
- Exhaustive experiments are conducted to demonstrate the effectiveness of the proposed HDR reconstruction. Two main aspects are explored: the computational cost required and the quality of the HDR output image, considering both quantitative and qualitative comparisons.

## 2. Related Works

A widely used approach is to reconstruct an HDR image from a single LDR image, which offers advantages such as the absence of misalignment and blur. However, this method introduces other types of defects known as artifacts [40]. Current methodologies attempt to address these artifacts using deep learning-based architectures, such as HDRUNet [17] and DCDR-UNet [41], which employ image restoration architectures based on multi-scale convolutions and Deformable Convolution Residual Blocks, respectively. Datasets developed for this type of reconstruction typically feature three input frames, as proposed by NTIRE [37] in 2021, but only one of these frames is used for training and testing the architectures.

Currently, the most widely used approach for HDR reconstruction involves creating an HDR image from LDR images with different exposures, known as multi-frame HDR reconstruction. Tursun et al. [42] identify ghosting artifacts as the main challenges in HDR reconstruction, including light-source motion, object movement, object deformation, and object occlusion. These challenges have led to the development of various feature extraction architectures and blocks based on CNN architectures [26,39], diffusion-based architectures [29], and innovative methods using Transformers [31,32]. A common theme among these methods is the extraction of features from multiple images and their fusion using image restoration architectures, achieving high-quality HDR reconstruction. Feature extraction is typically performed using alignment mechanisms [43] or attention blocks [44], where the central exposure image serves as a reference for applying the desired alignment or attention. The dataset commonly used in this type of reconstruction is the one proposed by Kalantari et al. [39] in 2017, though new proposals inspired by this dataset, such as the one by Tel et al. [32] in 2023, also exist.

Transformer-based models are widely used for HDR tasks due to their strong image reconstruction capabilities. The SwinIR architecture [45] was used by Liu et al. [31] in HDR-Transformer, adding a frame alignment mechanism before feature extraction. On the other hand, Kim et al. [46] used Transformers in a UNet form for feature alignment and refinement. Zou et al. [47] proposed HDT-HDR, which uses a dual Transformer mechanism that, in addition to performing self-attention on the input features, also performs refinement through convolutions and adds them at the end of the process, enriching the refinement. Chen et al. [48] presented a pyramid-shaped Transformer architecture called HFT-HDR for feature fusion, leaving the reconstruction task to a series of convolutional blocks. PASTA is an architecture proposed by Liu et al. [49] that performs multi-scale feature refinement using SwinIR-based Transformer layers in conjunction with channel-level attention. Yan et al. [50] proposed HyHDRNet, which consists of two networks: one focuses on removing ghost artifacts from features, and the other is a SwinIR-based network that enhances these features. Chi et al. [51] proposed SV-HDR, a novel technique using a Transformer architecture for denoising in a diffusion-based reconstruction approach, which outperforms other denoisers. Lastly, Yan et al. [52] proposed SSHDR, a semi-supervised reconstruction approach based on SwinIR using datasets with and without ground truth.

A methodology that has recently achieved excellent performance using only Transformer mechanisms is SCTNet, proposed by Tel et al. [32]. It adopts the SwinIR reconstruction architecture [45] and adds cross-attention mechanisms. The authors conducted comparative experiments using the Kalantari and Tel datasets, obtaining the best performance in HDR image reconstruction. The results were compared with proposals from Hu et al. [14] and Sen et al. [13], which are based on patch-level image fusion and use only CPUs for processing. For comparison purposes, the authors also used CNN-based architectures, including DHDRNet [39], which incorporates an alignment process for HDR reconstruction, DeepHDR [53], which aligns features using the medium-exposure image as a reference, and NHDRRNet [54], which uses non-local attention mechanisms instead of alignment to achieve the same purpose. Other architectures such as AHDRNet [55] use attention blocks for feature alignment and dense residual dilation blocks to reconstruct HDR images, while CEN-HDR [44] utilizes spatial attention to improve the extracted features with convolution mechanisms for further refinement.

Inspired by the generative approach, HDR-GAN [30] was proposed as the first architecture based on GANs for fusing multiple-exposure LDR images for HDR reconstruction. Evaluations using the Kalantari dataset demonstrated that this approach outperformed architectures such as Sen’s, Hu’s, and Kalantari’s architectures as well as DeepHDR, AHDRNet, and NHDRRNet under the same evaluation conditions. On the other hand, Diff-HDR [29] introduced a diffusion-based approach for HDR reconstruction, treating the HDR image as a conditional generative modeling task. Results on the Kalantari dataset showed good generalization for real-world images and competitive performance with state-of-the-art methodologies, including those of Hu and Kalantari as well as DeepHDR, AHDRNet, NHDRRNet, HDR-GAN, ADNet [27], APNT [56], ST-HDR [57], and HDR-Transformer [31]. In another approach, HDR-Transformer [31] replaced traditional convolutional feature encoding processes with Transformer-based attention mechanisms. The authors showed its superior performance compared to methods such as Sen’s, Hu’s, and Kalantari’s methods as well as DeepHDR, AHDRNet, NHDRRNet, HDR-GAN, and SwinIR [45]. They also compared inference time and model parameters, highlighting the superiority of their approach in balancing performance and efficiency.

The main focus of the HDR-GAN, DiffHDR, HDR-Transformer, and SCTNet architectures is on improving reconstruction quality. However, they do not prioritize the reduction of computational cost. As a result, Transformer-based approaches show a high associated computational cost, particularly during inference time when reconstructing an HDR image. In contrast to the state of the art, the architecture proposed in this paper primarily focuses on reducing computational cost without compromising the final HDR reconstruction quality. This approach is based on the Transformer mechanisms presented in SCTNet, where the number of original self-attention and cross-attention blocks is reduced and complemented with convolutional attention blocks to maintain reconstruction quality.

## 3. Proposed Method

This section is organized to address four key aspects of the proposed architecture. First, it describes the overall functioning of the architecture, explaining how the input image is processed and outlining the stages and blocks that make it up. Then, it provides a detailed explanation of the processing carried out by each block in the feature extraction stage, describing the CBAM block used in this stage and the concatenation of features after the convolution performed on each input frame. Next, this section discusses the feature refinement stage, where a Transformer-based architecture is used. The self-attention in the Global Self-Attention Block (G-SAB) and cross-attention in the Spatial Cross-Attention Block (S-CAB) applied to the input are explained, along with the additional feature fusion known as the skip connection. Finally, the loss function used during the training of the architecture is detailed, explaining the equations that make up this function.

### 3.1. Overall Architecture

The input LDR images are three-channel images (red, green, and blue). However, to enhance the efficiency of HDR reconstruction, the HDR equivalent for each frame is computed by applying gamma encoding, as described in Equation (1).

(1) $$H_{i}=\frac{L_{i}^{\gamma }}{t_{i}}$$

where $L_{i}\in R^{3\times H\times W}$ corresponds to each input frame i∈{1,2,3}; γ=2.2 is a typical value for gamma correction; $t_{i}$ is the exposure time of the frame used; and $H_{i}\in R^{3\times H\times W}$ is the result of applying gamma correction, which is still a three-channel image. Therefore, our input, denoted as $I_{i}\in R^{6\times H\times W}$, becomes a six-channel image, resulting from the concatenation of $L_{i}$ with $H_{i}$, as shown in Equation (2).

(2) $$I_{i}=\left[L_{i}\;H_{i}\right]$$

**Figure 1:**
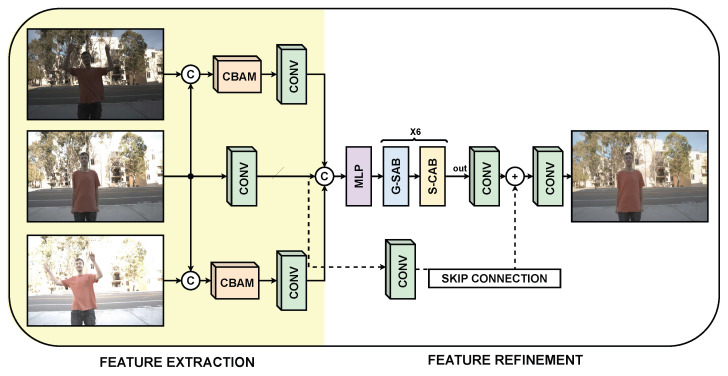
Proposed architecture for HDR reconstruction consisting of two stages; the first stage is feature extraction, and the second stage is feature refinement. The proposed architecture contains two CBAM blocks, representing consecutive channel-level attention and spatial-level attention; convolution blocks; and a Transformer architecture adopted from Tel et al. [32].

The proposed architecture is shown in Figure 1. The reconstruction process begins with the feature extraction stage, where three LDR input frames with different exposures are used. The exposure values may vary depending on the dataset, but the frame with the median exposure is always used as the reference for the spatial position of the objects. Next, both channel-level and spatial-level attention are applied using the CBAM block. This attention process highlights regions of the image, meaning it gives more importance to the areas that the model considers relevant in the high- or low-exposure frames with respect to the central frame.

Subsequently, a convolution block is applied to each frame to extract features from each image. These features are concatenated to be refined by a Transformer architecture. The next stage is feature refinement, which begins with an MLP block that enables the fusion of the non-linear features present in the input. This results in optimized features to be refined with the G-SAB block, which performs self-attention on these features, followed by the S-CAB block. The S-CAB block splits the features into groups of three to apply cross-attention and then concatenates them again at the end of this process.

The output of this refinement is filtered with a convolution and added to the initial features of the central frame in a process called skip connection. Finally, all the obtained features are decoded with a final convolution to reconstruct the corresponding HDR image.

### 3.2. Feature Extraction

#### 3.2.1. CBAM

**Figure 2:**
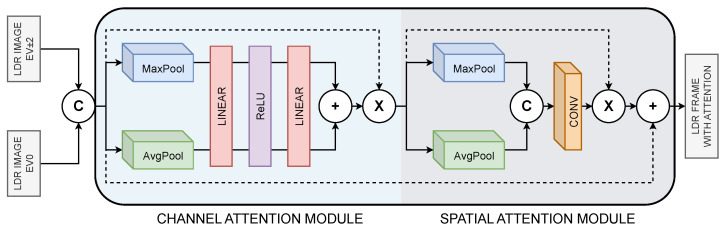
Composition of the CBAM block, consisting of two modules. The first one receives two concatenated images to apply channel-level attention, then applies spatial-level attention. The output is an image with both types of attention applied.

The frames $I_{1}$ and $I_{3}$ correspond to high- and low-exposure frames, respectively. The CBAM block takes as input one of these frames along with the reference frame $I_{2}$. Let the concatenation of both images be denoted as $F\in R^{12\times H\times W}$. In general, the attention process in CBAM can be described by Equation (3).

(3) $$\begin{array}{cc} F^{\prime } & =M_{C}\left(F\right)⊗F\\ F^{\prime \prime } & =M_{S}\left(F^{\prime }\right)⊗F^{\prime } \end{array}$$

where $M_{C}$ represents the channel-level attention, $M_{S}$ represents the spatial-level attention, $F^{\prime }$ is the output of the channel-level attention, and $F^{\prime \prime }$ is the output of the spatial-level attention. The symbol ⊗ denotes an element-wise operation. Figure 2 illustrates the process of the input image through the CBAM, which consists of the Channel Attention Module (CAM) and the Spatial Attention Module (SAM).

The CAM module generates an attention map that considers the channel-level relationships of the input features *F*. The attention map highlights the most important regions in the image. To achieve this, one channel is computed using Max Pooling and another using Average Pooling. Each channel is processed by an MLP with a ReLU activation function, where the MLP is shared by the two channels, meaning the same weights are used for channel-level attention. After this process, the two channels are summed to obtain the final attention map $M_{C}$, as described by Equation (4), where AvgPool refers to Average Pooling, MaxPool refers to Max Pooling, and σ represents the sigmoid function. This map is then multiplied by the input, generating a channel-attended image.

(4) $$M_{C}\left(F\right)=\sigma \left(\mathrm{MLP}\left(\mathrm{AvgPool}\left(F\right)\right)+\mathrm{MLP}\left(\mathrm{MaxPool}\left(F\right)\right)\right)$$

For the SAM module, where $F^{\prime }$ is an image with channel-level attention, the next step is to apply spatial-level attention. The first step is to calculate the Max Pooling and Average Pooling channels, which are then concatenated to generate a spatial attention map $M_{S}$ using a final convolution, as described in Equation (5). The map $M_{S}$ is then applied to the input image to obtain the corresponding spatial attention. Finally, this result is added to the initial input image, yielding an output image with both attentions applied.

(5) $$M_{S}\left(F^{\prime }\right)=\sigma \left(\mathrm{CONV}\left[\mathrm{AvgPool}\left(F^{\prime }\right)\;\mathrm{MaxPool}\left(F^{\prime }\right)\right]\right)$$

#### 3.2.2. Feature Concatenation

Herein, $F_{1}^{\prime \prime }$ represents the low-exposure image and $F_{3}^{\prime \prime }$ represents the high-exposure image after the CBAM block, while $I_{2}$ represents the medium-exposure image. The features of each of these images are extracted using a convolutional block and then concatenated to be used as inputs for the next stage. This process is mathematically described by Equation (6).

(6) $$z=[\mathrm{CONV}\left(F_{1}^{\prime \prime }\right)\;\mathrm{CONV}\left(I_{2}\right)\;\mathrm{CONV}\left(F_{3}^{\prime \prime }\right)]$$

### 3.3. Feature Refinement

#### 3.3.1. G-SAB

**Figure 3:**
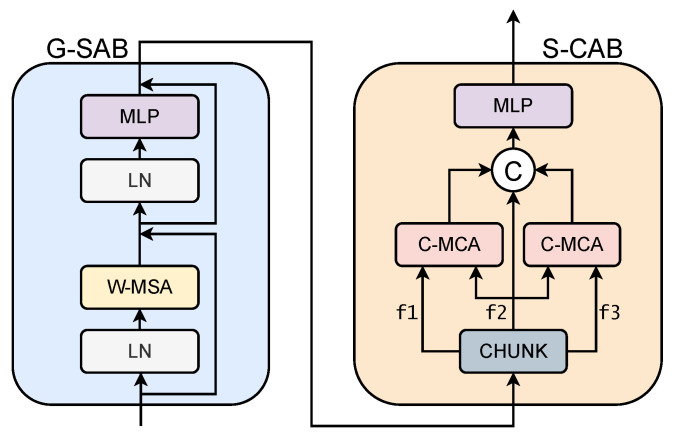
Two consecutive self-attention blocks. G-SAB applies conventional self-attention for the input features, while S-CAB applies cross-attention, using features of the central frame as the query and features of the other frames as the key and value.

To distinguish the spatial relationship between different features, a multi-head self-attention technique based on windows, as proposed by Tel et al. [32], was used. To achieve this, the image is divided into small patches of 8 × 8 pixels, which are tokenized to feed into the G-SAB block, as shown in Figure 3. Within this block, there are four main components: initially, the input patches are normalized by Layer Normalization (LN), followed by the calculation of patch-level self-attention in the W-MSA. The result is then normalized again with another LN, and non-linear relationships are found using an MLP. This makes it possible to assign importance to specific regions of the initial features in relation to the entire feature set. This modeling can be described by considering a patch sequence $z_{j-1}$ as input and $z_{j}$ as output, as mathematically expressed in Equation (7).

(7) $$\begin{array}{cc} \; & {\hat{z}}_{j}=W\text{-}\mathrm{MSA}\left(\mathrm{LN}\left(z_{j-1}\right)\right)+z_{j-1}\\ \; & z_{j}=\mathrm{MLP}\left(\mathrm{LN}\left({\hat{z}}_{j}\right)\right)+{\hat{z}}_{j} \end{array}$$

#### 3.3.2. S-CAB

To perform cross-attention given an input feature $z_{j}$, the feature is divided into three subgroups with an equal number of channels: $f_{1}$, $f_{2}$, and $f_{3}$, corresponding to the features of the low-, medium-, and high-exposure frames, respectively, as described in Equation (8).

(8) $$\left[f_{1}\;\;f_{2}\;\;f_{3}\right]=z_{j}$$

Next, channel multi-head cross-attention (C-MCA) is applied between these features, using $f_{2}$ as the query $q_{2}$; $f_{1}$ generates the key and value for $k_{1},v_{1}$, and $f_{3}$ generates the key and value for $k_{3},v_{3}$, respectively. Therefore, the output of the S-CAB can be written as shown in Equation (9), where $f_{12}^{\prime }$ and $f_{32}^{\prime }$ are the outputs after each C-MCA.

(9) $$\begin{array}{cc} f_{12}^{\prime } & =C\text{-}\mathrm{MCA}\left(q_{2},k_{1},v_{1}\right)=softmax\left(\frac{q_{2}\times k_{1}^{T}}{\sqrt{d_{k}}}\right)v_{1}\\ f_{32}^{\prime } & =C\text{-}\mathrm{MCA}\left(q_{2},k_{3},v_{3}\right)=softmax\left(\frac{q_{2}\times k_{3}^{T}}{\sqrt{d_{k}}}\right)v_{3} \end{array}$$

where $d_{k}$ is the scaling factor.

#### 3.3.3. Skip Connection

Considering out as the output of the Transformer network, there is a final convolution before the aggregation of the central features $F_{2}^{\prime }$ from the medium-exposure image, as given by Equation (10), where $\hat{H}$ represents the reconstructed HDR image.

(10) $$\hat{H}=\mathrm{CONV}\left(\mathrm{CONV}\left(F_{2}^{\prime }\right)+\mathrm{CONV}\left(out\right)\right)$$

### 3.4. Loss Function

Typically, HDR images are tone-mapped for visualization purposes, which is why it is common to calculate the loss function in the tone-mapped domain. For this, the commonly used μ-law mapping is applied, as described by Equation (11).

(11) $$T\left(H\right)=\frac{log(1+\mu H)}{log(1+\mu )},\;\;\;\mu =5000$$

where T(H) represents the tone-mapped image, *H* is the input HDR image, and μ is an intensity parameter. The loss function consists of two parts; the first part is the classic $L_{1}$ loss function, which calculates the mean absolute error (MAE), as shown in Equation (12), where $\hat{H}$ is the HDR image reconstructed by the network and *H* is the real HDR image.

(12) $$L_{1}={∥T\left(H\right)-T\left(\hat{H}\right)∥}_{1}$$

Additionally, another loss function $L_{p}$, called the perceptual loss, is calculated. This loss function is widely used in inpainting [58] for improving visual quality. It is computed using a pre-trained VGG-16 network, where the MAEs of the feature maps Φ calculated by VGG-16 are summed. This function is described by Equation (13).

(13) $$L_{p}={∥\Phi \left(T\left(H\right)\right)-\Phi \left(T\left(\hat{H}\right)\right)∥}_{1}$$

Thus, the final loss function L, as characterized in Equation (14), is composed of the two previously mentioned loss functions, resulting in a robust final function that considers errors not only at the pixel level but also at the feature level. A parameter α, set to 0.01, is used to adjust the value of the perceptual loss.

(14) $$L=L_{1}+\alpha \cdot L_{p}$$

## 4. Experiments and Results

### 4.1. Implementation Details

#### 4.1.1. Datasets

The training was carried out using the datasets from Kalantari [39] and Tel [32]. Kalantari’s dataset was selected because it is the most commonly used for HDR reconstruction tasks, and Tel’s dataset was selected to complement Kalantari’s. To overcome the challenge of limited training images, the image was divided into small patches of 128 × 128 pixels with a stride of 64 pixels. Data augmentation included rotation and image reflection. Additionally, separate training was performed for each dataset, resulting in two different models for comparison with the state of the art.

Tel: The dataset developed by Tel et al. [32] consists of 108 training scenes and 36 test scenes. This dataset was captured using a Nikon D700 (Nikon Corporation, Tokyo, Japan) camera, with up to nine different exposures taken to obtain the reference HDR image, and a tripod was used to ensure no movement during the capture. From the nine exposures, three images with different exposures are provided for each scene. As for the LDR images, they were captured by introducing small movements to simulate natural captures. The final dataset has a resolution of 1500 × 1000 pixels

Kalantari: In 2017, Kalantari et al. [39] developed a dataset that consists of 74 training scenes and 15 test scenes. The dataset was captured using a high-quality Canon EOS-5D Mark III (Canon Inc., Tokyo, Japan) camera. Initially, more than 100 scenes were captured in RAW format with a resolution of 5760 × 3840 pixels; they were later resized to a resolution of 1500 × 1000 pixels. During the capture process, small intentional movements between frames were introduced to simulate misalignment. This dataset utilizes three input images with different exposures.

Based on Tel et al. [32], images from the Kalantari test set exhibit a high proportion of overexposed pixels. This introduces challenges not directly related to HDR reconstruction, arising from prolonged exposure times in the reference image, minimal time differences between the exposures of the LDR images, and the limitation that three input images may be inadequate for this dataset. In contrast, Tel’s test set does not present these particular issues.

#### 4.1.2. Experimental Setup

The proposed network was implemented using PyTorch 2.4.0 with Python 3.11. The ADAM optimizer was used with a fixed learning rate of $2\times 10^{-4}$, $\beta_{1}$ set to 0.9, $\beta_{2}$ set to 0.999, and ϵ set to $10^{-8}$. The network training was carried out in the PAVIC laboratory’s data center, utilizing two Nvidia HGX A100 GPUs (Nvidia Corporation, Santa Clara, CA, USA) with 40 GB of memory, a batch size of 32, and 150 training epochs. The training took approximately three days.

#### 4.1.3. Evaluation Metrics

The performance evaluation was carried out by comparing the quality metrics PSNR and SSIM, both in the linear domain and in the logarithmic domain, denoted by *l*-PSNR, μ-PSNR, *l*-SSIM, and μ-SSIM. The notation *l* represents the calculation in the linear domain, i.e., the reconstructed HDR image compared with the real HDR reference, while μ represents the calculation in the mapped domain, using Equation (11) to map the reconstructed HDR image and the HDR reference image. HDR-VDP-2, a metric specifically developed to quantify quality in HDR images, was also used.

Peak Signal-to-Noise Ratio: PSNR is a metric proposed by Huynh et al. [59] that quantitatively determines the quality of an image by assessing the level of noise between two signals, measured in decibels (dB). A value of 0 dB indicates that the signals are completely different, while a higher value indicates less distortion between the two signals. Mathematically, PSNR is given by Equation (15).

(15) $$\mathrm{PSNR}=10\cdot \log_{10}\left(\frac{1}{\mathrm{MSE}}\right)$$

The MSE is described by Equation (16), which calculates the squared difference between the values of two signals. In the case of images, it measures the squared difference of the pixel values, where *y* is the original image or ground truth and $\hat{y}$ is the reconstructed image.

(16) $$\mathrm{MSE}=\frac{1}{n}\sum_{i=0}^{n}{\left(y_{i}-\hat{y_{i}}\right)}^{2}$$

A high PSNR indicates that the reconstruction is of high quality. However, there are cases where visually similar images may have a low PSNR, as simple pixel errors might not adequately capture the structural similarity.

Structural Similarity Index Measure: The SSIM metric, proposed by Sara et al. [60], measures the structural similarity between an image *x* and another image *y*. Mathematically defined by Equation (17), it calculates the average pixel values for both images, denoted as $\mu_{x}$ and $\mu_{y}$, respectively. The variances $\sigma_{x}^{2}$ and $\sigma_{y}^{2}$, as well as the covariance $\sigma_{xy}$ between the two images, are also computed. The constants $c_{1}$ and $c_{2}$ are defined as $c_{1}={\left(k_{1}L\right)}^{2}$ and $c_{2}={\left(k_{2}L\right)}^{2}$, with $k_{1}=0.01$, $k_{2}=0.03$, and $L=2^{\mathrm{bits}\;\mathrm{per}\;\mathrm{pixel}}-1$.

(17) $$\mathrm{SSIM}\left(x,y\right)=\frac{\left(2\mu_{x}\mu_{y}+C_{1}\right)\left(2\sigma_{xy}+C_{2}\right)}{\left(\mu_{x}^{2}+\mu_{y}^{2}+C_{1}\right)\left(\sigma_{x}^{2}+\sigma_{y}^{2}+C_{2}\right)}$$

HDR-VDP-2: Using deep learning-based algorithms, Mantiuk et al. [61] introduced a model capable of discriminating quality between two HDR images, taking into account real-world conditions such as luminance, dynamic range, and color levels. The HDR-VDP-2 model was trained using the LIVE and TID2008 datasets to assess image quality, acknowledging that the comparison of HDR images involves both pixel value measurements and the properties of the display panel. The authors assume the characteristics of a standard LCD screen for this purpose, demonstrating through rigorous experimentation that HDR-VDP-2 was calibrated and reliably predicts HDR image quality by quantifying visual differences into a numerical value.

### 4.2. Evaluation and Comparison

A comparative evaluation was conducted using the Tel [32] and Kalantari [39] datasets. The analysis considered prominent state-of-the-art architectures based on GANs, diffusion, and Transformers, including HDR-GAN [30], Diff-HDR [29], HDR-Transformer [31], and SCTNet [32].

#### 4.2.1. Quantitative Analysis

For the quantitative evaluation using the Tel dataset, it was necessary to train the Diff-HDR and HDR-GAN architectures to compute the metrics. The metrics for the HDR-Transformer and SCTNet architectures were obtained from SCTNet [32]. The results are presented in Table 1.

In terms of μ-PSNR, μ-SSIM, and *l*-SSIM, the proposed architecture achieved the best results, followed by SCTNet. The results for *l*-PSNR and HDR-VDP-2 place the proposed method in second place, as it was outperformed by SCTNet. On the other hand, HDR-Transformer ranks as the third best in performance. Thus, the results obtained with this dataset demonstrate that the proposed architecture delivers the best performance with minimal distortion and better structural similarity between the reconstructed image and its reference.

For the evaluation using the Kalantari dataset, the values of the metrics obtained by Tel et al. [32] and Diff-HDR [29] are considered. The results are shown in Table 2.

The results show the superiority of SCTNet in almost all metrics. When considering the image quality metric for reconstructed images under real-world conditions, HDR-VDP-2, the proposed methodology outperforms all other architectures.

#### 4.2.2. Qualitative Analysis

A detailed visual comparison was performed on images from the Tel [32] and Kalantari [39] datasets, specifically using scenes 29 and 34 from the Tel dataset and scenes 9 and 7 from the Kalantari dataset. Each of the analyzed scenes will be presented with images arranged in three columns: the input LDR images, the reconstructed image, and the area for detailed analysis.

**Figure 4:**
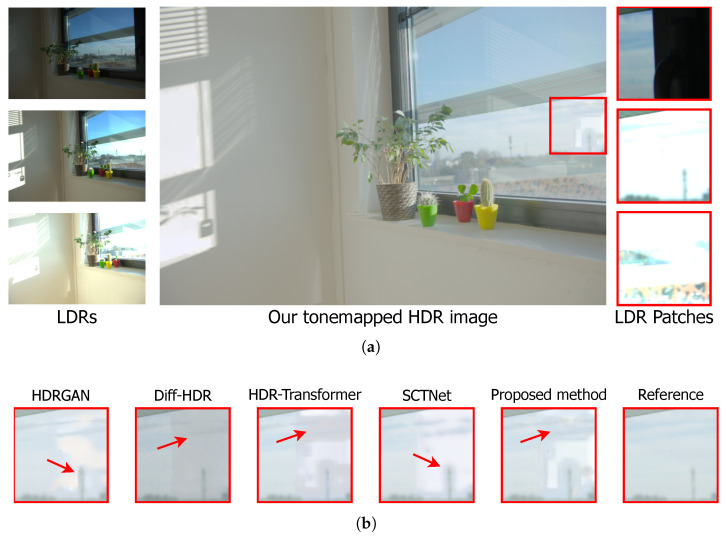
Qualitative comparison of the reconstruction of scene 29 from the Tel dataset [32]. (**a**) Input images with different exposures and the HDR image reconstructed by the proposed method, with the analyzed region highlighted by a red box. (**b**) HDR image reconstructions by HDR-GAN [30], Diff-HDR [29], HDR-Transformer [31], SCTNet [32], and the proposed methodology, followed by the reference image. The red arrows indicate the main differences in the images.

Scene 29 from the Tel dataset is presented in Figure 4a, where the three input LDR images, the HDR image reconstructed by the proposed method, and the area for detailed analysis are shown. The selected area, highlighted by a red box, exhibits overexposure in the input LDR images, making it a challenging scene to reconstruct.

Figure 4b shows the areas of interest cropped from the images reconstructed by the analyzed architectures. Due to the complexity of the scene, the presence of ghosting artifacts becomes evident in all the reconstructions, with all architectures facing difficulties in performing the reconstruction. Additionally, HDR-GAN and SCTNet exhibit a blur effect observed in the vertical structure, indicated by an arrow. Diff-HDR and HDR-Transformer show a darker tone than the reference. In contrast, the reconstruction by the proposed method produces the closest color to the reference while also reducing the intensity of the ghosting artifact.

**Figure 5:**
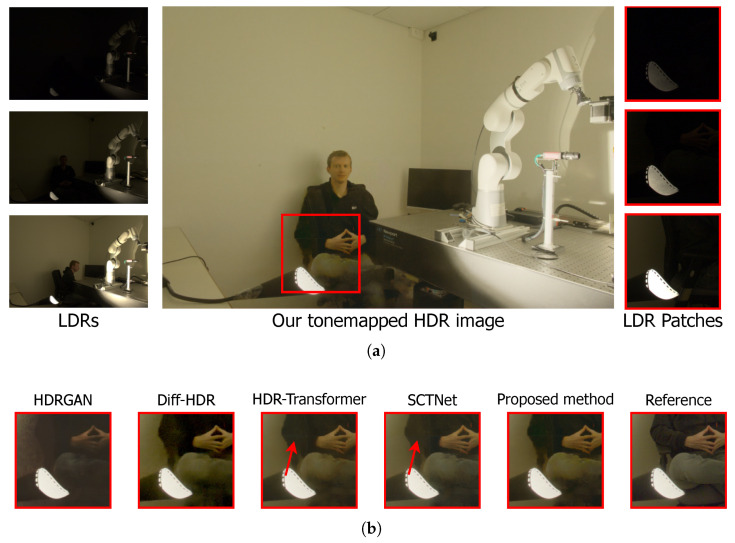
Qualitative comparison of the reconstruction of scene 34 from the Tel dataset [32]. (**a**) Input images with different exposures and the HDR image reconstructed by the proposed method, with the analyzed region highlighted by a red box. (**b**) HDR image reconstructions by HDR-GAN [30], Diff-HDR [29], HDR-Transformer [31], SCTNet [32], and the proposed methodology, followed by the reference image. The red arrows indicate the main differences in the images.

Similarly, Figure 5a illustrates scene 34 from the Tel dataset, where the selected area for analysis is highlighted by a red box. This area presents a challenge for reconstruction due to the lack of information in the input LDR images caused by underexposure, as observed in the LDR patches.

Figure 5b presents the analysis areas extracted from the images reconstructed by each architecture. The reconstruction performed by HDR-GAN shows color discrepancies compared to the reference. The result achieved by Diff-HDR exhibits inconsistencies in its reconstruction, presenting a significant amount of noise. HDR-Transformer and SCTNet show similar characteristics regarding the blur effect, which is more intensely observed in the region indicated by the red arrow. On the other hand, the results obtained by the proposed method demonstrate a reconstruction with more appropriate color contrast than the reference.

**Figure 6:**
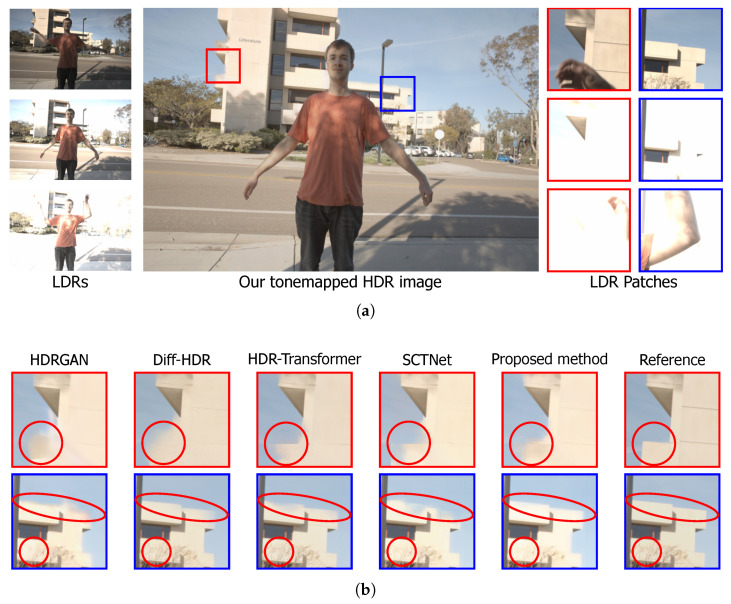
Qualitative comparison of the reconstruction of scene 9 from the Kalantari dataset [39]. (**a**) Input images with different exposures and the reference HDR image, with red and blue boxes highlighting the analyzed regions. (**b**) HDR image reconstructions by HDR-GAN [30], Diff-HDR [29], HDR-Transformer [31], SCTNet [32], and the proposed methodology, followed by the reference image. The red circles highlight the main challenges in the reconstructed images.

Figure 6a illustrates scene 9 from the Kalantari dataset, a figure with overexposure of objects that poses a challenge for HDR reconstruction. Two regions of this scene were analyzed, with the areas marked by the red box and the blue box highlighting regions where the information in the different LDR frames is scarce and where there is high contrast in the edges and walls of the building, showing a complex scenario for reconstruction.

The reconstruction behaviors of the different analyzed architectures for the demarcated areas are presented in Figure 6b. In the HDR reconstruction for the red-marked region containing building walls with a sky background, HDR-GAN and Diff-HDR architectures show more difficulty in reconstructing details of the analyzed physical structure, as highlighted within the red circle. In contrast, HDR-Transformer, SCTNet, and the proposed method achieves better reconstruction of the building walls in the same region. In the blue-marked region, the red details show that the roof of the building and the tree branches in the reconstructed images are inadequately captured in the HDR-GAN and Diff-HDR reconstructions compared to the reference image. However, HDR-Transformer, SCTNet, and the proposed method presents better reconstructions of these details. These results highlight the efficiency of Transformer-based architectures for HDR reconstruction compared to others.

**Figure 7:**
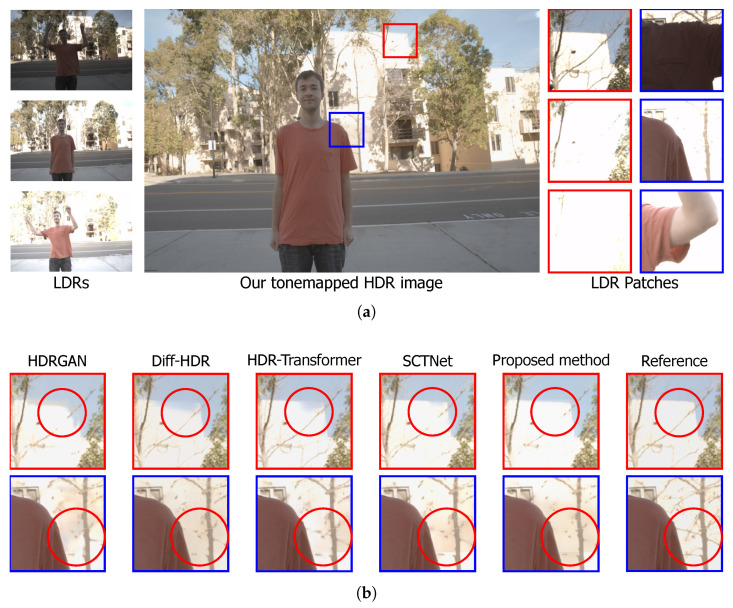
Qualitative comparison of the reconstruction of scene 7 from the Kalantari dataset [39]. (**a**) Input images with different exposures and the reference HDR image, with red and blue boxes highlighting the analyzed regions. (**b**) HDR image reconstructions by HDR-GAN [30], Diff-HDR [29], HDR-Transformer [31], SCTNet [32], and the proposed methodology, followed by the reference image. The red circles highlight the main challenges in the reconstructed images.

Finally, scene 7 from the Kalantari dataset is analyzed, as shown in Figure 7a, where the regions of interest are marked in red and blue. Visual results highlight the challenge of preserving features in a scenario with a varied background, where it is necessary to maintain the attributes of the tree branches over the building and the outlines of the building edges with the sky. Given this, it can be verified that in the image recovery shown in the circle within the red-marked region in Figure 7b, HDR-GAN and Diff-HDR face difficulties in reconstructing the building edges. HDR-Transformer and SCTNet exhibit the same behavior but to a lesser extent. On the other hand, the proposed method successfully addresses this problem by coherently recovering the building edges. However, when observing the tree branches, the models face difficulties in their reconstruction, as can be corroborated in the circles within the blue-marked region, which may characterize a challenge in the reconstruction process for such scenarios.

#### 4.2.3. Comparison of Computational Cost

A comparative analysis of computational cost was conducted, with metrics including the number of parameters, the number of multiply—accumulate (MAC) units, the size of the trained model, and the inference time. As Diff-HDR is executed on a GPU, the results for all architectures were calculated using an Nvidia HGX A100 40 GB GPU. The results obtained by the proposed methodology were compared to results achieved by other methodologies analyzed, as presented in Table 3.

The results presented in Table 3 demonstrate that the proposed architecture is significantly lighter than the others, as it contains only one-third of the parameters of SCTNet, which ranks as the second-best model, reducing the number of parameters by 0.7 million. Additionally, the proposed method requires three times fewer MAC operations for reconstruction, resulting in a reduction of 170.59 GMAC compared to SCTNet, the second fastest. Similarly, the proposed method has a more compact model size than the second-smallest model, HDR-GAN, by 20.1 MB. Regarding inference time, the proposed approach achieves lower latency than most of the other architectures, being surpassed only by HDR-GAN. Consequently, the results indicate that the proposed model significantly improves computational cost compared to other state-of-the-art methodologies, as supported by an inference time that is 4.09 s faster than that of the SCTNet model.

### 4.3. Ablation Study

The importance of the blocks used in the proposed architecture in this paper was demonstrated through an ablation study. For this study, the architecture was divided into four key components: baseline, CBAM, SCT, and skip connection. This study helps to assess the contribution of each component to the overall performance and efficiency of the model, enabling a better understanding of the impact of each block on the HDR reconstruction task.

Baseline: Considering Figure 1, this component refers to all the convolutional blocks used in the architecture, with the exception of the convolution employed in the Skip connection.

CBAM: These blocks are used to apply channel attention and spatial attention to the input frames. They highlight the important regions within the frames to enhance feature extraction from these areas.

SCT: It combines two key components: G-SAB, which applies Transformer self-attention, and S-CAB, which applies cross-attention. Together, these components enhance the features extracted from the input frames.

Skip connection: This component involves the addition of the central frame features with those refined by the Transformer blocks, serving to retain the central image as a reference before decoding the features.

To highlight the importance of the complete architecture, a training was first conducted using only the baseline. The CBAM block was then added, followed by a second training. Subsequently, the SCT block was incorporated, and a third and final training was performed. The results for PSNR and SSIM from these trainings were calculated and are presented in Table 4.

The results suggest that each block has a positive impact on the proposed HDR reconstruction architecture, improving the outcomes obtained in each metric, with the complete architecture achieving the best reconstruction quality. The components with the greatest impact on the architecture are CBAM and SCT, which significantly enhance the results, and the skip connection also has a positive impact, albeit to a lesser extent.

## 5. Conclusions

This paper proposes a Transformer-based approach for HDR reconstruction, focusing on achieving high-quality images with low computational complexity. The methodology incorporates a reduced number of self-attention blocks for feature refinement, enhancing them in an initial stage using the CBAM attention module. For comparison and validation purposes, extensive experiments were conducted, considering both quantitative and qualitative evaluations using the Kalantari and Tel datasets. Based on a quantitative comparison of the PSNR, SSIM, and HDR-VDP-2 metrics, the obtained results demonstrate that the proposed architecture significantly outperforms other state-of-the-art methodologies in terms of HDR reconstruction quality. Regarding the challenges of qualitative comparison, the results highlight the excellent performance of the proposed approach in reconstructing image regions with high contrast in complex scenarios where limited information is available. Moreover, a comparison of the proposed methodology with other Transformer-based models, evaluated in terms of computational cost for the same HDR reconstruction task, reveals an improvement of 0.7 million parameters, 170.59 GMAC, 20.1 MB, and 4.09 s. Consequently, the proposed methodology achieves a reduction of approximately 65% in the computational cost required to perform the entire reconstruction process. Additionally, the significance of the complete architecture is emphasized through an ablation study of its main components. Finally, this approach presents a promising direction for the development of Transformer-based models with low computational cost and efficient performance in HDR reconstruction tasks.

## Figures and Tables

**Table 1:** Comparative table of image quality metrics for architectures using the Tel dataset.

Method	μ-PSNR ↑	*l*-PSNR ↑	μ-SSIM ↑	*l*-SSIM ↑	HDR-VDP-2 ↑
HDR-GAN [30]	40.32	44.35	0.9833	0.9913	67.89
Diff-HDR [29]	42.33	46.79	0.9849	0.9948	68.91
HDR-Transformer [31]	42.39	46.35	0.9844	0.9948	69.23
SCTNet [32]	42.55	**47.51**	0.9850	0.9952	**70.66**
**Proposed method**	**43.16**	47.46	**0.9876**	**0.9956**	69.76

Note: The best result is shown in **bold**, and the second best is underlined. ↑ indicates that higher values are better.

**Table 2:** Comparative table of image quality metrics for architectures using the Kalantari dataset.

Method	μ-PSNR ↑	*l*-PSNR ↑	μ-SSIM ↑	*l*-SSIM ↑	HDR-VDP-2 ↑
HDR-GAN [30]	43.92	41.57	0.9905	0.9865	65.45
Diff-HDR [29]	44.11	41.73	0.9911	0.9885	65.52
HDR-Transformer [31]	44.32	42.18	0.9916	0.9884	66.03
SCTNet [32]	**44.49**	**42.29**	**0.9924**	**0.9887**	66.65
**Proposed method**	44.10	42.07	0.9917	0.9886	**67.79**

Note: The best result is shown in **bold**, and the second best is underlined. ↑ indicates that higher values are better.

**Table 3:** Comparison of computational cost between the proposed architecture and state-of-the-art models. Results were calculated using an Nvidia HGX A100 40 GB GPU.

Method	Par. (M) ↓	MAC (G) ↓	Size (MB) ↓	Inf. (s) ↓
HDR-GAN [30]	2.63	778.61	10.6	**0.19**
Diff-HDR [29]	75.13	—	289.0	178.16
HDR-Transformer [31]	1.22	981.81	53.4	7.61
SCTNet [32]	0.99	255.54	28.0	6.28
**Proposed method**	**0.29**	**84.95**	**7.9**	2.19

Note: The best result is shown in **bold**, and the second best is underlined. ↓ indicates that lower values are better.

**Table 4:** Results of an ablation study of the proposed architecture in terms of HDR reconstruction quality using the Kalantari dataset.

Base	CBAM	SCT	Skip c.	μ-PSNR ↑	*l*-PSNR ↑	μ-SSIM ↑	*l*-SSIM ↑
✔	✘	✘	✘	27.05	28.68	0.9518	0.9440
✔	✔	✘	✘	36.16	36.30	0.9818	0.9777
✔	✔	✔	✘	44.03	41.97	0.9915	0.9880
✔	✔	✔	✔	**44.10**	**42.07**	**0.9917**	**0.9886**

Note: ✔ means the block is present and ✘ means the block was removed from the architecture. The best result is in **bold**. ↑ indicates that higher values are better.

## Data Availability

The data presented in this study are available on request from the corresponding author. The data are not publicly available due to privacy restrictions.
